# Reply to Hamzavi et al. Comment on “Islam et al. Helminth Parasites among Rodents in the Middle East Countries: A Systematic Review and Meta-Analysis. *Animals* 2020, *10*, 2342”

**DOI:** 10.3390/ani13223467

**Published:** 2023-11-10

**Authors:** Md Mazharul Islam, Elmoubashar Farag, Mohammad Mahmudul Hassan, Devendra Bansal, Salah Al Awaidy, Abdinasir Abubakar, Hamad Al-Romaihi, Zilungile Mkhize-Kwitshana

**Affiliations:** 1 Department of Animal Resources, Ministry of Municipality and Environment, Doha P.O. Box 35081, Qatar; 2 School of Laboratory Medicine and Medical Sciences, College of Health Sciences, University of KwaZulu Natal, Durban 4000, South Africa; 3 Ministry of Public Health, Doha P.O. Box 42, Qatar; dbansal@moph.gov.qa (D.B.); halromaihi@moph.gov.qa (H.A.-R.); 4 Faculty of Veterinary Medicine, Chattogram Veterinary and Animal Sciences University, Chattogram 4225, Bangladesh; miladhasan@yahoo.com; 5 Ministry of Health, P.O. Box 393, PC 113 Muscat, Oman; salah.awaidy@gmail.com; 6 Infectious Hazard Preparedness (IHP) Unit, WHO Health Emergencies Department (WHE), World Health Organization, Regional Office for the Eastern Mediterranean, Cairo 11371, Egypt; abubakara@who.int; 7 School of Life Sciences, College of Agriculture, Engineering & Science, University of KwaZulu Natal, Durban 40000, South Africa; mkhizekwitshanaz@ukzn.ac.za; 8 South African Medical Research Council, Cape Town 7505, South Africa

Rodents serve as an important reservoir or carrier of zoonotic pathogens on a global scale. When we began to investigate the risks of such zoonoses in Qatar, we discovered a knowledge gap regarding rodent-borne zoonoses in the Middle East. Consequently, we decided to update the relevant information and identify the gaps that require attention to emphasize research in these areas. Therefore, our aforementioned review [1] represents the first work of its kind in this region, where we compiled published articles spanning over 50 years in the Middle East.

We sincerely appreciate the interest shown by Hamzavi et al. [2] in our article [1] and their comprehensive review of it. In response to their comments, we would like to address a few points. Firstly, it is important to distinguish between estimated pooled prevalence (EPP) and true prevalence (TP), as they represent distinct concepts in determining prevalence. EPP is derived from meta-analysis, providing a summary measure or estimation of prevalence by considering findings from multiple sources. However, it is subject to limitations due to variations in study designs, populations, sample sizes, and data collection methods. On the other hand, TP is determined using population-based studies or statistical methods such as percentile calculation. While meta-analysis can inform us about the overall prevalence based on existing evidence, it does not directly provide the true prevalence [3,4]. For example, the true crude fatality rate due to MERS is 35% [5], whereas the meta-analysis yielded a rate of 18.49% [6].

Throughout our review [1], we conducted meticulous and diligent observation and analysis to ensure the accuracy of the presented information. Our primary objective was to retrieve precise and relevant data while avoiding any misinformation. In cases where an article did not explicitly provide the required information, we made every effort to extract the necessary data by thoroughly examining the entire article.

Now, let us delve into the article mentioned by Hamzavi et al. [2], authored by Nateghpour et al. [7]. Upon thorough examination, it becomes evident that the study involved testing 100 rodents belonging to different species. Table 2 provides clear information indicating that out of the tested rodents, a total of 47 were found to be infected with various species of parasites. Specifically, 11 *Tatera indica* were infected with *Hymenlopis diminuta*, and 8 *Tatera indica* were infected with *Hymenlopis nana*. If we consider the count of positive rodents, the value is indeed 47. Notably, the article did not mention any instances of coinfection in the results. If there had been any coinfection, they would not have mentioned “Total: 47”, or the total number of infected rodents would have been less than 47. In such a scenario, we can exclude the results of Nateghpour et al. from the meta-analysis. As the article does not report any coinfection, we cannot disregard its results in the meta-analysis by assuming the possibility of coinfection. Therefore, our observation of a total of 19 rodents infected with helminths remains valid. While it may appear that Hamzavi et al.’s observation contradicts ours, even if we consider our observation to be incorrect, this discrepancy would not significantly impact the estimated pooled prevalence.

We believe that our response effectively addresses the concerns raised by Hamzavi et al. [2]. We are open to arranging an online meeting to further discuss this issue, address any remaining knowledge gaps, and explore potential areas of research collaboration. We kindly request the editorial team of “MDPI Animals” to assist in organizing such a meeting, inviting Hamzavi et al. [2] and any other researchers who are interested in this field of study.
